# Molecular and Phenotypic Evaluation of Antibiotic Resistance in Enteric Rods Isolated from the Oral Cavity

**DOI:** 10.3390/antibiotics14060564

**Published:** 2025-05-31

**Authors:** Yineth Neuta, Natalia Leguizamon, Paula Pajaro, Manuela Zarate, Mauricio Julio, Manuela Pantoja, Isabella Llerena, Nathaly Andrea Delgadillo

**Affiliations:** 1 Unidad de Investigación Básica Oral-UIBO, Vicerrectoría de Investigaciones, Facultad de Odontología, Universidad El Bosque, Bogotá 110121, Colombia; 2 Facultad de Odontología, Universidad El Bosque, Bogotá 110121, Colombia

**Keywords:** enteric rods, oral cavity, genes, antibiotic resistance

## Abstract

Gram-negative enteric rods (GNERs) are transient members of the oral microbiota and are considered a superinfection in patients with periodontitis that poses local and systemic risks due to associations with infections and multidrug resistance, including extended-spectrum beta-lactamases. These pathogens often resist antibiotics such as amoxicillin, doxycycline, and ciprofloxacin, complicating dental treatments. Though their resistance patterns vary, links between specific resistance genes and phenotypic resistance remain unclear. **Objectives**: To determine the correlation between resistance genes (*blaTEM*, *blaSHV*, *tetQ*, *tetM*, *qnrB*, *qnrS*, and *mph*(A)) and phenotypic resistance in GNERs isolated from oral cavity samples. **Methods**: A total of 90 oral isolates of GNERs were isolated from patients in a dental clinic, and bacteria were identified by the BD BBL Crystal biochemical panel. The antibiotic susceptibility testing was conducted through broth microdilution following CLSI standards for drives such as amoxicillin, amoxicillin/clavulanic acid, doxycycline, ciprofloxacin, and azithromycin. Resistance genes, including *blaTEM*, *blaSHV*, *tetQ*, *tetM*, *qnrS*, *qnrB*, and *mph*(A), were detected using polymerase chain reaction and gel electrophoresis. The proportions of species, resistance genes, and minimum inhibitory concentration values were statistically analyzed. **Conclusions**: As expected, most enteric bacteria showed natural resistance to beta-lactams. Significant resistance to azithromycin was observed in some species. Genotypic and phenotypic profiles suggest the existence of alternative resistance mechanisms; therefore, other mechanisms associated with antibiotic resistance should be investigated.

## 1. Introduction

The Gram-negative enteric rods (GNERs) included in the family *Enterobacteriaceae* are commonly found in the microbiota of the gastrointestinal tract because they are natural inhabitants of soil and water. Though usually thought to be nonresidents of the oral microbiota, they can often be part of the transient microbiota, depending on the ingestion of water or food from contaminated sources or poor personal hygiene [1]. In patients with periodontitis, enteric rods are considered a superinfection [2], a multifactorial, chronic inflammatory disease characterized by dysbiotic microbiota and the loss of periodontal tissue support [3]. These pathogens can survive in the subgingival pocket, even after periodontal treatment such as debridement and surgery, contributing to refractory periodontitis, oral mucosal infections, and dental implant failure, particularly in patients who are immunosuppressed, diabetic, xerostomic, or elderly [4,5].

Recent studies have reported the frequency of enteric bacteria isolated from patients with periodontitis, observing a low frequency in Germany (5%) [2], whereas another study reported the presence of enteric bacteria in 45.9% of patients with periodontitis in Brazil [6]. Colonization of GNERs in the oral cavity poses local and systemic risks because it is associated with serious diseases such as pneumonia, meningitis, systemic infections, endocarditis, and urinary tract infections.

Furthermore, GNERs often harbor determinants of antibiotic resistance, including extended-spectrum beta-lactamases (ESBLs), aggravating public health concerns. The existence of multidrug-resistant GNER strains in the oral cavity of healthy humans highlights the importance of epidemiological surveillance to reduce their spread and associated health risks [7]. Antimicrobial resistance (AMR) is embedded in the immediate public health problem, as it is driven by indiscriminate antimicrobial overuse, which are frequently used during mechanical and surgical treatment alternatives in dentistry [2]. GNERs often show either intrinsic or acquired resistance to first-line antibiotics such as beta-lactams and metronidazole [8]. Alternatives to these drugs, such as fluoroquinolones (ciprofloxacin), macrolides (azithromycin and clarithromycin), and tetracyclines (doxycycline), have shown limited capacity to act because of the establishment of new resistance mechanisms [9].

Recent work has shown regional variations in the NRG resistance patterns. For example, in Brazil, *Klebsiella pneumoniae*, *Escherichia coli*, and *Enterobacter aerogenes* isolated from dentures showed resistance to amoxicillin and amoxicillin/clavulanic acid, and some isolates were resistant to tetracycline [10]. Another study detected the resistance of *Enterobacteriaceae* to other classes of antimicrobial agents including tetracycline (25%), sulfamethoxazole/trimethoprim (12.5%), and chloramphenicol (18.8%). No resistance to quinolones, ciprofloxacin, or norfloxacin was detected among the enteric rods isolated from the periodontal pockets [11]. In Nigeria, high frequencies of beta-lactamase genes (*blaOXA*, *blaCTX*-M, and *blaSHV*) have been observed in *Enterobacteriaceae* isolated from patients with periodontitis [12]. In Europe, Basic et al. processed oral samples from 211 Swedish patients with mucosal lesions, periodontitis, or peri-implantitis, identifying a total of 138 isolates of enteric rods, which showed high resistance to antibiotics commonly used in dentistry (amoxicillin, amoxicillin + clavulanic acid, ampicillin, clindamycin, doxycycline, erythromycin, oxacillin, penicillin V, and tetracycline); most of them were susceptible to ciprofloxacin [13]. In Poland, GERs constituted 37.4% of bacteria in oral samples from 182 healthy adolescents, 27.5% of which were resistant to multiple antibiotics such as ampicillin (100%), ceftazidime (69.1%), meropenem (60.3%), gentamicin (60.3%), piperacillin/tazobactam (52.9%), piperacillin (45.6%), amikacin (38.2%), ciprofloxacin (36.8%), trimethoprim/sulfamethoxazole (36.8%), and imipenem (29.4%) [7]. In Asia, the presence of bacteria with antibiotic resistance to third-generation cephalosporins and carbapenems, recovered from the oral cavity of patients between 40 and 90 years old, has been reported, particularly GERs resistant to cephalosporins and carbapenems in 19.5% of patients. The main genera and species found were *Acinetobacter* (39.7%), *Stenotrophomonas* (24.4%), *Pseudomonas* (14.5%), *Enterobacter* spp., (3.8%), and *E. coli* (3.1%). In *E. coli*, the presence of ESBL genes has been reported [14]. In a multicenter study conducted in Colombia, enteric bacilli were observed in 29.8% of all patients, both periodontally healthy and diseased individuals; however, their resistance patterns have not been well examined [15]. Despite this progress, the relationship between specific resistance genes and phenotypic sensitivity to antibiotics used in dentistry, namely, amoxicillin, amoxicillin/clavulanic acid, doxycycline, ciprofloxacin, and azithromycin, remains poorly understood. Therefore, the aim of this study is to determine the correlation between resistance genes (*blaTEM*, *blaSHV*, *tetQ*, *tetM*, *qnrB*, *qnrS*, and *mph*(A)) and phenotypic resistance in Gram-negative *Enterobacteriaceae* isolated from oral cavity samples.

## 2. Results

### 2.1. Frequency of Enteric Rods in Oral Isolates

In the oral cavity samples studied here, 90 bacterial isolates of different enteric species were found. The dominant species was *Enterobacter cloacae* with 28.9% (*n* = 26), followed by *Klebsiella oxytoca* with 21.1% (*n* = 19) and *Cronobacter sakazakii* with 15.6% (*n* = 14); subsequently, we found *Serratia marcescens* (14.4%; *n* = 13), *Klebsiella pneumoniae* (6.7%; *n* = 6), *Serratia liquefaciens* (6.7%; *n* = 6), *Enterobacter gergoviae* (5.6%; *n* = 5), and *Klebsiella aerogenes* (1.1%; *n* = 1) (Table 1). These findings reveal the variety of bacterial species in the oral cavity and the predominance of *Enterobacter cloacae*, which suggests its capacity to adapt and colonize the oral cavity.

### 2.2. Evaluation of Antibiotic Susceptibility in Oral Isolations of Enteric Rods

Different patterns of antibiotic resistance were observed (Table 2). Overall, 97.8% (*n* = 88) of species were resistant to β-lactam antibiotics such as amoxicillin (AMX), and 91.1% (*n* = 82) were resistant to amoxicillin/clavulanic acid (AMC). Additionally, 53.3% (*n* = 48) of isolates were resistant to azithromycin (AZT), 18.8% (*n* = 17) were resistant to doxycycline (DO), and 1.1% were resistant to ciprofloxacin (CIP). For sulfonamides, 1.4% (*n* = 1) of isolates were resistant to trimethoprim/sulfamethoxazole (TMP/SMX). We observed that 10% (*n* = 9) of the enteric bacterial isolates were simultaneously resistant to more than two antibiotics, and only one isolate was sensitive to all the antibiotics evaluated. In the 90 isolates evaluated (MIC90), the inhibition range was 8–32 µg/mL for AMX; the minimum inhibitory concentration 50 (MIC50) was 32 µg/mL, while the MIC90 was 32 µg/mL. For AMC, the inhibition range was 4–32 µg/mL, the MIC50 was 32 µg/mL, and the MIC90 was 32 µg/mL. For AZT, an inhibition range of 2–64 µg/mL was observed, the MIC50 was <32 µg/mL, and the MIC90 was 64 µg/mL. For DO, the inhibition range was 0.5–32 µg/mL, the MIC50 was 4 µg/mL, and the MIC90 was 8 µg/mL. For CIP, the inhibition range was 0.0004–32 µg/mL, the MIC50 was 0.0312 µg/mL, and the MIC90 > 0.125 µg/mL. For TMP/SMX, the inhibition range was <0.25–8 µg/mL, the MIC50 was >0.25 µg/mL, and the MIC90 > 0.25 µg/mL (Table 2).

**Figure 1:**
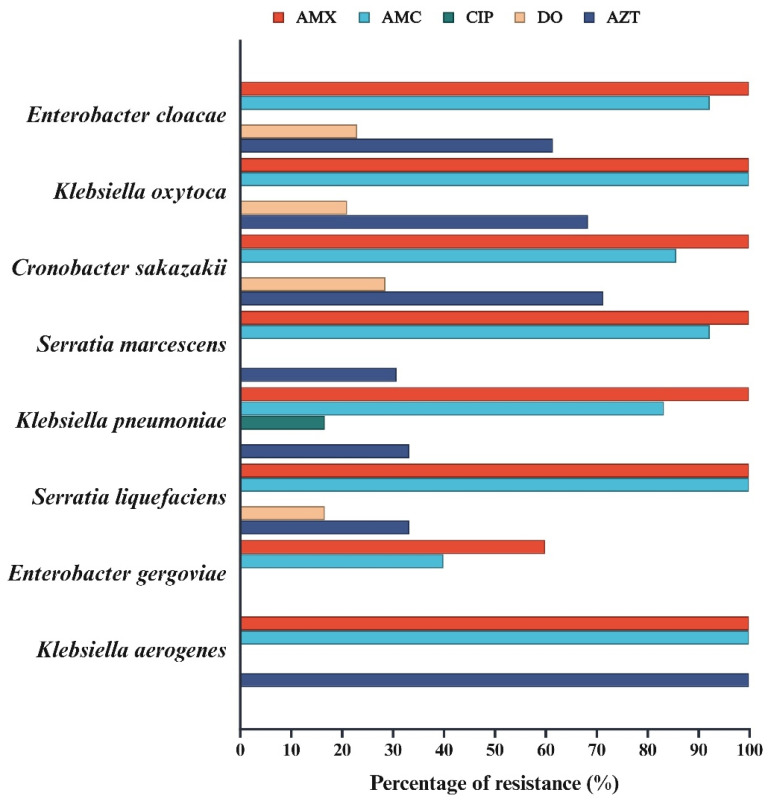
Percentage of resistance to different antibiotics across several bacteria evaluated in this study. The antibiotics analyzed were amoxicillin (AMX), amoxicillin/clavulanic acid (AMC), ciprofloxacin (CIP), doxycycline (DO), and azithromycin (AZT) for the species evaluated. High resistance to AMX and AMC and minimal to no resistance to CIP was noted in all species.

Resistance varied across species. In particular, *Enterobacter cloacae* showed 100% resistance to AMX, 92.3% to AMC, 23.1% to DO, 61.5% to AZT, and no resistance to CIP. *K. oxytoca* showed 100% resistance to AMX and AMC, 21.1% to DO, 68.4% to AZT, and no resistance to CIP. *C. sakazakii* showed 100% resistance to AMX, 85.7% to AMC, 28.6% to DO, 71.4% to AZT, and no resistance to CIP. *Serratia marcescens* showed 100% resistance to AMX, 92.3% to AMC, 30.8% to AZT, and no resistance to DO or CIP. *K. pneumoniae* showed 100% resistance to AMX, 83.3% to AMC, 16.7% to CIP, 33.3% to AZT, and no resistance to DO. *S. liquefaciens* showed 100% resistance to AMX and AMC, 16.7% to DO, 33.3% to AZT, and no resistance to CIP. *Enterobacter gergoviae* showed the lowest levels of resistance: 60% to AMX; 40% to AMC; and no resistance to CIP, DO, or AZT. *Klebsiella aerogenes* distinguished itself by its 100% resistance to AMX, AMC, and AZT, with no resistance to CIP or DO. Generally, high resistance to AMX and AMC was observed, whereas resistance to CIP was low (Figure 1). Table 3 summarizes the ranges and MIC50 and MIC90 values for each bacterium.

### 2.3. Prevalence of Resistance Genes in Oral Isolates of Enteric Rods

Table 4 shows the frequency of the resistance genes in the 90 isolates. Genes associated with resistance to β-lactam antibiotics (*blaTEM* and *blaSHV*), tetracyclines (*tetM* and *tetQ*), fluoroquinolones (*qnrB* and *qnrS*), and macrolides (*mph*(A)) were detected in the isolates of enteric rods. The most frequently detected genes in the isolates were *tetM* (51.1%), *blaTEM* (26.6%), *qnrB* (17.7), and *blaSHV* (14.4%). Low levels of *tetQ* and *qnrS* were detected (1.1% each), while *mph*(A) was not detected in any of the species.

*E. cloacae* showed the highest frequency of *tetM* (57.7%), followed by *blaTEM* (2.1%) and *blaSHV* (80.8%). While *qnrB* (1%) and *tetQ* (3.8%) were detected in a lower proportion, neither *qnrS* nor *mph*(A) was detected. *K. oxytoca* showed a high frequency of *tetM* (52.6%), *blaTEM* (31.6%), and *qnrB* (36.8%). The detection of *blaSHV* was 21.1%, whereas *tetQ*, *qnrS*, and *mph*(A) were not detected. *C. sakazakii* showed predominance of the *tetM* gene (64.3%), followed by *blaTEM* (14.3%) and *blaSHV* (14.3%). *qnrB* (21.4%) was present, whereas *tetQ*, *qnrS*, and *mph*(A) were not detected. *S. marcescens* was present with *blaTEM* with a frequency of 30.8%. *blaSHV* (15.4%) and *tetM* (23.1%) were also detected, and *qnrB* was detected in 15.4% of isolates; *tetQ*, *qnrS*, and *mph*(A) were not observed. *K. pneumoniae* showed significant frequencies of *tetM* (66.7%), *blaTEM* (33.3%), *blaSHV* (16.7%), and *qnrS* (16.7%). *tetQ*, *qnrB*, and *mph*(A) were not detected. *S. liquefaciens* showed only *tetM* (16.7%). *E. gergoviae* showed high frequencies of *tetM* (80%) and *blaTEM* (20%). *K. aerogenes* showed *blaTEM* and *blaSHV* with a frequency of 100%, while other genes were not detected.

### 2.4. Genotype–Phenotype Relationship

**Figure 2:**
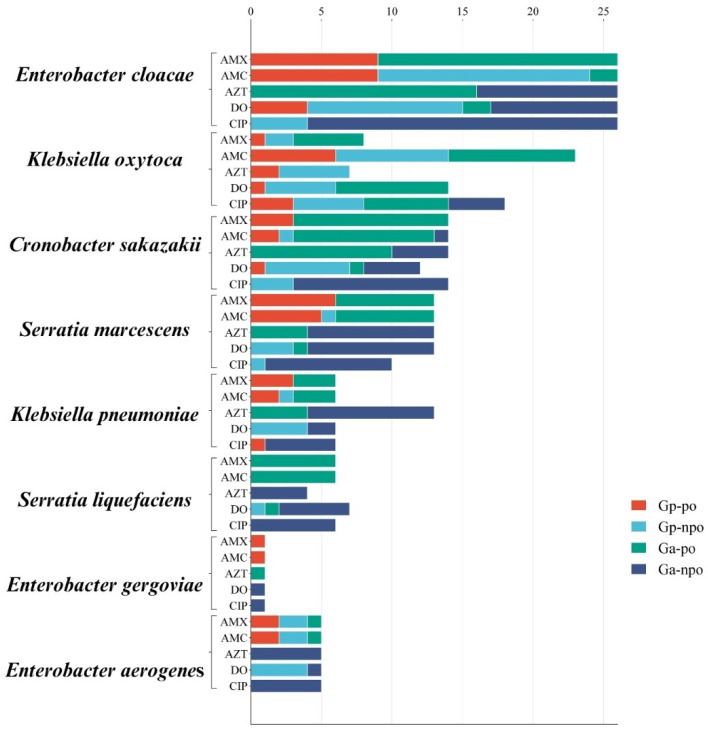
Molecular and phenotypic evaluation of antibiotic resistance in enteric rods. Gp-po: gene present and phenotype observed; Gp-npo: gene present but no phenotype observed; Ga-po: gene absent and phenotype observed; Ga-npo: gene absent and no phenotype observed.

Of the 90 isolates of enteric bacteria analyzed, 89 were resistant to beta-lactams, and 32 isolates harbored resistance genes. The relationship between genotype and phenotype showed notable variability across bacterial species and antibiotics (Figure 2). The connection between ARGs and the observed phenotypes was investigated, classifying the profiles into the following categories: Gp-po (gene present and resistance observed), Gp-npo (gene present but resistance not observed), Ga-po (gene absent and resistance observed), and Ga-npo (gene absent and resistance not observed). *E. cloacae* demonstrated high susceptibility to β-lactams; for amoxicillin, 9 isolates were categorized as Gp-po and 17 as Gp-npo, whereas 9 Gp-po and 15 Ga-po were found for amoxicillin/clavulanic acid. These results suggest alternate resistance mechanisms. Most isolates were sensitive to azithromycin (Gp-npo: 16, Ga-npo: 10), whereas the resistance rates of doxycycline and ciprofloxacin were not discrete (Ga-po: 11 and 4, respectively).

*K. oxytoca* showed moderate resistance to β-lactams, with amoxicillin classified as Gp-po in 1 and Ga-po in 2; conversely, amoxicillin/clavulanic acid was classified as Gp-po in 6 and Ga-po in 8. Moderate resistance to azithromycin was observed (Gp-po: 2, Ga-po: 5); in contrast, the remaining 2 Gp-po and 5 Ga-po isolates demonstrated resistance to ciprofloxacin. *C. sakazakii* exhibited lower resistance to β-lactams compared with other species, particularly with amoxicillin showing 3 Gp-po and 11 Gp-npo, and amoxicillin/clavulanic acid showing 2 Gp-po, 10 Gp-npo, and 1 Ga-po. Azithromycin and ciprofloxacin resistance was minimal in most isolates in the Gp-npo and Ga-npo profiles, respectively. Substantial resistance was noted against amoxicillin (Gp-po: 6, Gp-npo: 7) and amoxicillin/clavulanic acid (Gp-po: 5, Ga-po: 1) for *S. marcescens*. Azithromycin resistance provided a low evidence base (Ga-npo: 9), remaining nonetheless in the low resistance range for doxycycline (Ga-po: 3) and ciprofloxacin (Ga-po: 1). *K. pneumoniae* displayed much less resistance, with amoxicillin showing a mild response for 3 Gp-po and 3 Gp-npo, and amoxicillin/clavulanic acid being less significant, with only 2 Gp-po and 1 Ga-po. The isolates most susceptible to these antibiotics included azithromycin (Gp-npo: 4, Ga-npo: 9) and ciprofloxacin (Ga-npo: 5). *S. liquefaciens* was consistently susceptible to antibiotics immediately; for both azithromycin and ciprofloxacin, all isolates were still in Ga-npo. Doxycycline resistance was represented by only one isolate as Ga-po. Resistance against *E. gergoviae* was uncommon, with a single Gp-po isolate for the β-lactams and other isolates classified as Ga-npo for most other antibiotics. *K. aerogenes* showed moderate resistance to β-lactams (Gp-po: 2, Ga-po: 2); similarly, all isolates were susceptible to azithromycin and ciprofloxacin (Ga-npo).

*E. cloacae* and *K. oxytoca* had high β-lactam resistance, mostly present as Gp-po and Ga-po. Less resistant *C. sakazakii* and *S. marcescens* had mixed profiles, with occasional resistance to β-lactams and doxycycline. Lower-resistant *K. pneumoniae*, *S. liquefaciens*, *E. gergoviae*, and *K. aerogenes* showed a higher sensitivity profile, with Ga-npo being the most predominant, with occasional resistance without any detected genes (Ga-po).

## 3. Discussion

GNERs are transient colonizers of the oral cavity; their presence can increase the risk of systemic dissemination and serious infections, such as pneumonia, bacteremia, and endocarditis, especially in patients with immunosuppression, diabetes, advanced age, xerostomia, or other oral disorders. Furthermore, their ability to carry antibiotic resistance genes and resist various antibiotics is a significant therapeutic challenge [1].

In this study, the most prevalent bacteria identified were *E. cloacae* (28.9%) and *K. pneumoniae* (21.1%), which agrees with Goldberg et al. in 1997 [17], who associated these microorganisms with the production of compounds such as volatile sulfides and cadaverine related to bad breath. Likewise, in 2007, Botero et al. [18] described these same species as the most frequently isolated in patients with chronic periodontitis, suggesting a possible relationship between their colonization and the development of chronic periodontal disease. In 2024, Basi et al. [13] also reported these species as the most frequently isolated from patients with oral inflammatory dysbiotic conditions. In the Colombian population, Mayorga et al. [19] reported frequencies of *Klebsiella*, *Enterobacter*, and *Serratia* of 10–15.2%. The presence of these microorganisms in the oral cavity has been associated with poor oral hygiene or the consumption of contaminated food and water [1].

Almost all identified *Enterobacteriaceae* showed high resistance to beta-lactams. This study revealed that more than 90% of the isolates were resistant to amoxicillin and amoxicillin/clavulanic acid, reflecting low sensitivity to commonly used antibiotics in dental practice. Such resistance is related to intrinsic resistance mechanisms [2], associated with chromosomally encoded OXY-like beta-lactamases and mutations in the *blaOXY1* and *blaOXY2* promoters, which drive the overproduction of these enzymes [20]. Despite excluding patients with recent antibiotic use, resistance determinants such as plasmids and transposons exacerbate the clinical challenges caused by these bacteria. These findings underscore the urgent need for the rational use of antimicrobials in dentistry to mitigate global resistance.

For azithromycin, a high percentage of resistance was observed in oral isolates, which is consistent with Nguyen et al. [21], who found a high prevalence of bacteria resistant to this antibiotic group, mediated by the expression of different genes that inhibit its action mechanism; however, a significant percentage of sensitive enteric rods was also observed. Azithromycin resistance has been associated with various genes such as *mph*(A). However, in the present study, *mph*(A) was not detected in any of the isolates, which contrasts with Gomes et al. [22], who detected this gene in 40 of 43 isolates of enteric rods. Pawlowski et al. [23] also found *mph*(A) in *Escherichia* (50%), *Klebsiella* (37.5%), and *Enterobacter* (6.1%). Therefore, other genes associated with macrolide resistance, such as *term*, *msr*, *cml*, and *la* [24], should be evaluated.

Most isolates were susceptible to DO and CIP, which is consistent with other studies [25] that measured the prevalence and antimicrobial susceptibility of GNERs isolated from subgingival biofilm, finding high rates of susceptibility to CIP and low resistance to DO (25%) in species such as *Serratia* spp., *Klebsiella* spp., and *Enterobacter* spp. Although most isolates were sensitive to CIP, *qnrB* was found in 17.7% of them. This gene has been frequently found in enteric bacteria such as *Klebsiella* and *Enterobacter*. A significant frequency of *tetM* was also detected (51%), though resistance to DO was low. Tetracycline resistance genes are the most commonly found in the mouth, and *tetM* is the most frequently reported gene [6]. *Enterobacteriaceae* are considered an important gene reservoir. However, while some resistance genes may not be associated with a resistant phenotype, they can be transferred to other species or expressed under appropriate conditions [26].

This study revealed variability in genotype–phenotype relationships, highlighting the complexity of AMR mechanisms among different bacterial species and the antibiotics used. Limitations of this study include lack of knowledge of some diagnoses associated with isolates and the patient history of exposure to antibiotics. However, the categorization of the isolates into four profiles (Gp-po: gene present/resistance observed, Gp-np: gene present/resistance not observed, Ga-po: gene absent/resistance observed, and Ga-npo: gene absent/resistance not observed) helped show patterns of expected and unexpected resistance mechanisms that will guide future studies on GERs and antibiotic resistance in dental patients.

## 4. Materials and Methods

### 4.1. Sample Collection and Identification

Enteric rods were isolated from 90 oral isolates that were part of strains maintained by the oral microbiology laboratory of the UIBO Institute (Basic Oral Research Unit) collected from the saliva of patients who attended the dental clinics of El Bosque University, of whom 47.8% were diagnosed with periodontitis. Samples were preserved in BHI broth (Oxoid CM1135B, Basingstoke, UK) +10% glycerol at −80 °C, thawed, and plated on MacConkey agar (Oxoid CM10115, Basingstoke, UK) and incubated at 37 °C for 24 h. Subsequently, each genus and species were identified using the BD BBL Crystal Enteric/Nonfermented ID System biochemical panel (Becton Dickinson, Franklin Lakes, NJ, USA). Isolates were then stored in molecular-grade water at −20 °C for DNA extraction and conventional polymerase chain reaction (PCR).

### 4.2. Antibiotic Susceptibility Testing by Microdilution in Broth

Sterile 96-well round-bottom plates were prepared by adding 50 μL of cation-adjusted Müeller–Hinton broth (CAMBH, Becton Dickinson, Franklin Lakes, NJ, USA) to each well. The antibiotics were previously prepared as follows: amoxicillin, amoxicillin/clavulanic acid, doxycycline, ciprofloxacin, and azithromycin, according to CLSI 2022 [16]. Serial dilutions of the antibiotics were prepared from the highest to the lowest concentrations. Subsequently, dilutions of the inocula were added from the isolates of interest, which were previously adjusted in saline solution in a spectrophotometer until reaching a turbidity of 0.5 McFarland scale (625 nm OD: 0.08–0.13) to obtain a concentration equivalent to 1.5 × 10^8^ CFU/mL.

The MIC50 and MIC90 of the isolates were determined. All plates that met the controls; purity control of the CAMBH broth; growth control; and control of the strains *Escherichia coli* ATCC^®^ 25922, *Enterococcus faecalis* ATCC^®^ 29212, or *Staphylococcus aureus* ATCC^®^ 29213 with the antibiotics were validated according to the ranges indicated by the CLSI [16].

### 4.3. Detection of Antibiotic Resistance Genes by PCR

DNA was extracted by thermal shock to detect resistance genes in the enteric rod isolates. The presence of *blaTEM*, *blaSHV*, resistance to beta-lactams, *tetQ*, *tetM*, resistance to doxycycline, *qnrS*, *qnrB*, resistance to ciprofloxacin, and *mph*(A) resistance to macrolides was detected by conventional PCR with the primers and protocols previously reported in the literature (Table 5) with some modifications. Positive and negative controls were included.

PCR products were visualized by agarose gel electrophoresis at a concentration of 1.5% in Tris-acetate-EDTA buffer with 0.5 μg/mL of ethidium bromide in a transilluminator (Gel Doc-BioRad, Hercules, CA, USA) with ultraviolet light at 300 nm.

### 4.4. Statistical Analysis

Databases were built in Microsoft Office Professional Plus Excel 2016 and used to calculate *Enterobacteriaceae* frequencies, species frequencies, resistance genes, and antibiotic resistance (MIC50 and MIC90) to statistically analyze the results.

## 5. Conclusions

As expected, most enteric bacteria showed natural resistance to beta-lactams. Significant resistance to azithromycin was observed in some species, although it was not associated with the presence of resistance genes; therefore, other mechanisms related to antibiotic resistance should be investigated.

This work highlighted the high prevalence of GNERs in the oral cavity and their association with periodontitis, which increases the risk of systemic dissemination in vulnerable patients. Significant multi-resistance to the most used antibiotics in dentistry, especially β-lactams and macrolides, poses a considerable therapeutic challenge, especially when resistance genes such as *tetM*, *blaTEM*, and *qnrB* are present.

The variable correlation between genotypic and phenotypic profiles found here suggests the existence of alternative resistance mechanisms or rare conditions for the expression of these genes in specific niches of the oral cavity; this further highlights the need for ongoing epidemiological surveillance and the rational use of antibiotics during periodontal treatment, as well as investigations of other genetic determinants and new therapeutic alternatives that will help curb the growing threat of AMR in the context of oral and systemic health.

## Figures and Tables

**Table 1:** Species of *enteric rods* (*n* = 90) isolated from the oral cavity of patients in clinics between 2007 and 2019.

Organisms	*n*	Frequency (%)
*Enterobacter cloacae*	26	28.9
*Klebsiella oxytoca*	19	21.1
*Cronobacter sakazakii*	14	15.6
*Serratia marcescens*	13	14.4
*Klebsiella pneumoniae*	6	6.7
*Serratia liquefaciens*	6	6.7
*Enterobacter gergoviae*	5	5.6
*Klebsiella aerogenes*	1	1.1

**Table 2:** Global resistance and minimum inhibitory concentration (MIC) ranges, MIC50, and MIC90.

Antibiotic	*n*	Range ^1^	MIC50 ^2^	MIC90 ^3^	R %
AMX	90	8–32	32	32	97.8
AMC	90	4–32	32	32	91.1
AZT	90	2–>64	32	64	53.3
DO	90	0.5–32	4	8	18.8
CIP	90	0.0004–32	0.0312	0.125	1.1
TMP/SMX	68	<0.25–8	<0.25	<0.25	1.4

AMX: amoxicillin; AMC: amoxicillin/clavulanic acid; AZT: azithromycin; DO: doxycycline; CIP: ciprofloxacin; TMP/SMX: trimethoprim-sulfamethoxazole. ^1^ Antimicrobial range in which inhibition was detected (µg/mL); ^2^ MIC for 50% of the isolates; ^3^ MIC for 90% of isolates; R: resistance range (µg/mL) according to the CLSI 2022 [16] for each antibiotic evaluated.

**Table 3:** MIC ranges and MIC50 and MIC90 values for the antimicrobial drugs tested here.

Antibiotic	AMX	AMC	AZT	CIP	DO
*Enterobacter cloacae*					
Range ^1^	32	8–16	4–64	0.004–0.25	0.5–16
MIC50 ^2^	32	32	32	0.156	2
MIC90 ^3^	32	32	32	0.312	8
*Klebsiella oxytoca*					
Range ^1^	16–32	16–32	16–64	0.007–0.25	1–32
MIC50 ^2^	32	32	32	0.0625	4
MIC90 ^3^	32	32	64	0.125	8
*Cronobacter sakazakii*					
Range ^1^	32	8–16	4–>64	0.004–0.125	0.5–32
MIC50 ^2^	32	32	4	0.156	4
MIC90 ^3^	32	32	32	0.25	32
*Serratia marcescens*					
Range ^1^	32	4–32	4–64	0.004–0.125	0.5–8
MIC50 ^2^	32	32	16	0.0312	2
MIC90 ^3^	32	32	64	0.125	4
*Klebsiella pneumoniae*					
Range ^1^	32	4–32	2–64	0.0312–32	1–4
MIC50 ^2^	32	32	16	0.0625	4
MIC90 ^3^	32	32	32	0.125	4
*Serratia liquefaciens*					
Range ^1^	32	32	2–32	0.004–0.0625	0.5–16
MIC50 ^2^	32	32	16	0.0156	0.5
MIC90 ^3^	32	32	32	0.0625	2
*Enterobacter gergoviae*					
Range ^1^	8–32	8–32	8–16	0.007–0.125	2–8
MIC50 ^2^	32	32	8	0.0156	4
MIC90 ^3^	32	32	16	0.0312	4
*Klebsiella aerogenes*					
Range ^1^	32	32	32	0.125	4
MIC50 ^2^	32	32	32	0.125	4
MIC90 ^3^	32	32	32	0.125	4

^1^ Antimicrobial range in which inhibition was detected (µg/mL); ^2^ MIC for 50% of the isolates; ^3^ MIC for 90% of the isolates.

**Table 4:** Frequency of *blaTEM*, *blaSHV*, *tetM*, *tetQ*, *qnrB*, *qnrS*, and *mph*(A) in 90 oral isolates of bacterial species evaluated in this study.

Organism	*blaTEM* F (%)	*blaSHV* F (%)	*tetM* F (%)	TetQ F (%)	*qnrB* F (%)	*qnrS* F (%)	*mph*(A) F (%)
*Enterobacter cloacae*	8 (2.1)	3 (80.8)	15 (57.7)	1 (3.8)	4 (1)	0	0
*Klebsiella oxytoca*	6 (31.6)	4 (21.1)	10 (52.6)	0	7 (36.8)	0	0
*Cronobacter sakazakii*	2 (14.3)	2 (14.3)	9 (64.3)	0	3 (21.49)	0	0
*Serratia marcescens*	4 (30.8)	2 (15.4)	3 (23.1)	0	2 (15.4)	0	0
*Klebsiella pneumoniae*	2 (33.3)	1 (16.7)	4 (66.7)	0	0	1 (16.7)	0
*Serratia liquefaciens*	0	0	1 (16.7)	0	0	0	0
*Enterobacter gergoviae*	1 (20)	0	4 (80)	0	0	0	0
*Klebsiella aerogenes*	1 (100)	1 (100)	0	0	0	0	0
Total	24 (26.6)	13 (14.4)	46 (51.1)	1 (1.1)	16 (17.7)	1 (1.1)	0

**Table 5:** Primer sequences for detecting resistance genes.

Gene	Sequence (5′–3′)	Amplicon Size (bp)	Reference
*mph*(A)	GTGAGGAGGAGCTTCGCGAG TGCCGCAGGACTCGGAGGTC	403	[21]
*blaSHV*	TCGTTATGCGTTATATTCGCC GGTTAGCGTTGCCAGTGCT	868	[27]
*blaTEM*	ATGAGTATTCAACATTTCCG CCAATGCTTAATCAGTGAGG	858	[28]
*tetM*	GACACGCCAGGACATATGG TGCTTTCCTCTTGTTCGAG	397	[29]
*tetQ*	GGCTTCTACGACATCTATTA CATCAACATTTATCTCTCTG	755	[30]
*qnrS*	GGAAACCTACAATCATACATA GTCAGGATAAACAACAATACC	657	[31]
*qnrB*	GACAGAAACAGGTTCACCGGT CAAGACGTTCCAGGAGCAACG	594	[31]

## Data Availability

The original contributions presented in this study are included in the article. Further inquiries can be directed to the corresponding author(s).
